# An Internet‐based platform for the estimation of outcrossing potential between cultivated and Chilean vascular plants

**DOI:** 10.1002/ece3.2854

**Published:** 2017-03-14

**Authors:** Pablo Cid, Carlos Aguirre, Miguel Ángel Sánchez, Daniel Zamorano, Maritza Mihoc, Erika Salazar, Gustavo Chacón, Humberto Navarrete, Marcelo Rosas, Humberto Prieto

**Affiliations:** ^1^ Biotechnology LaboratoryLa Platina Research StationInstituto de Investigaciones AgropecuariasLa PintanaSantiagoChile; ^2^ Asociación Gremial ChileBio CropLifeProvidenciaSantiagoChile; ^3^ Limnology LaboratoryFacultad de CienciasUniversidad de ChileMacul, Santiago de ChileChile; ^4^ Institute of Ecology and BiodiversityFacultad de CienciasUniversidad de ChileMacul, Santiago de ChileChile; ^5^ Genetic Resources Unit and Germplasm BankLa Platina Research StationInstituto de Investigaciones AgropecuariasLa PintanaSantiagoChile; ^6^ Computer Sciences LaboratoryLa Platina Research StationInstituto de Investigaciones AgropecuariasLa PintanaSantiagoChile; ^7^ Molecular Fruit Phytopathology LaboratoryFacultad Ciencias AgropecuariasUniversidad de ChileLa PintanaSantiagoChile; ^8^ Genetic Resources Unit and Germplasm BankIntihuasi Research StationInstituto de Investigaciones AgropecuariasVicuñaChile

**Keywords:** Chile, cultivated species, introduced species, native species, outcrossing potential, vascular flora

## Abstract

A national‐scale study of outcrossing potential within Chilean vascular flora was conducted using an upgraded algorithm, which adds parameters such as pollinator agents, climate, and geographic conditions. Datasets were organized and linked in a Web platform (www.flujogenico.cl), in which the development of a total outcrossing potential (TOP) predictor was formulated. The TOP predictor is the engine in the Web platform, which models the effect of a type of agricultural practice on others (coexistence calculation mode) and on the environment (biodiversity calculation mode). The scale for TOP results uses quintiles in order to define outcrossing potential between species as “very low,” “low,” “medium,” “high,” or “very high.” In a coexistence analysis considering 256 species (207 genera), the 10 highest TOP values were for genera *Citrus*,* Prunus*,* Trifolium*,* Brassica*,* Allium*,* Eucalyptus*,* Cucurbita*,* Solanum*,* Lollium*, and *Lotus*. The highest TOP for species in this analysis fell at “high” potential, 4.9% of the determined values. In biodiversity mode, seven out of 256 cultivated species (2.7%) were native, and 249 (97.3%) corresponded to introduced species. The highest TOP was obtained in the genera *Senecio*,* Calceolaria*,* Viola*,* Solanum*,* Poa*,* Alstroemeria*,* Valeriana*,* Vicia*,* Atriplex*, and *Campanula*, showing “high” potential in 4.9% of the values. On the other hand, 137 genetically modified species, including the commercial and pre‐commercial developments, were included and represented 100 genera. Among these, 22 genera had relatives (i.e., members of the same genus) in the native/introduced group. The genera with the highest number of native/introduced relatives ranged from one (*Ipomea*,* Limonium*,* Carica*,* Potentilla*,* Lotus*,* Castanea*, and *Daucus*) to 66 species (*Solanum*). The highest TOP was obtained when the same species were coincident in both groups, such as for *Carica chilensis*,* Prosopis tamarugo*, and *Solanum tuberosum*. Results are discussed from the perspective of assessing the possible impact of cultivated species on Chilean flora biodiversity. The TOP predictor (http://epc.agroinformatica.cl/) is useful in the context of environmental risk assessment.

## 1 Introduction

Technology adoption in agriculture allows for new horizons in productivity and quality. The involved crops must satisfy several regulatory issues to be approved for seed multiplication, and they are constantly subjected to economic, quality, and environmental impact analyses (ISF, 2014). In the same way, genetically modified (GM) crops could be released into the environment, and the inclusion of this technology must deal with technical capacities to accomplish regulatory requirements on each country (French, 2010).

Concerns about the environmental impact and coexistence of different crops focus on the premise that an eventual gene flow could potentially result in the loss of genetic purity or the introduction of undesired traits into wild relatives. Gene flow depends on variables such as the specific crop, its location, the presence of sexually compatible relatives, and the advantages of a specific trait (Armstrong, Fitzjohn, & Newstrom, 2005; Flannery, Meade, & Mullins, 2005; Whitney, Ahern, Campbell, Albert, & King, 2010). Other factors are also critical, such as the amount of pollen produced, longevity, dispersal (by wind, animals, or insects), plant/weed density, and the dormancy/rehydration of pollen (Whitney et al., 2010).

Special attention has been given to evaluation, measurement, and modeling of gene flow and its influence over cultivated species, wild relatives, and conventional agriculture (Fuchs & Gonsalves, 1999; ISF, 2014). The impact of agriculture on landscapes where crops are introduced has already been substantially analyzed, including GM crops (Sánchez et al., 2016). From the perspective of vascular plants, both GM and conventional agriculture (including organic practices) can be considered to impact niches in which they are used. This is of particular relevance when high biodiversity areas (zones in which high endemism is developed) are close to agriculturally relevant spots, such as in the Mediterranean zone located in Chile between parallels 25°S and 40°S and the coast strip up to 19.5°S, which are considered among the 25 hotspots for high biodiversity under conservation priority (Myers, Mittermeier, Mittermeier, & da Fonseca, 2000). These areas coincide with centers of origin for three cultivated species: strawberry (*Fragaria chiloensis*), bromus grasses (*Bromus* sp.), and potato (*Solanum tuberosum*). For this latter, whereas its center of origin and diversity is located at the Peruvian Andes, areas in Chile have been also recognized as secondary center of origin for this species and its wild relatives (Contreras et al., 1992).

Recently, a database composed of cultivated (conventional and GM), introduced, and native species in Chile has been used to predict the outcrossing potential between them and 11 relevant GM crops worldwide. This includes those that are commercially available and those with potential to be attractive for Chilean farmers (Sánchez et al., 2016). Although no significant impact on local flora was predicted for most of them, there is evidence for a need for a more comprehensive prediction platform that includes crucial factors such as pollinator insects, geographic conditions of landscapes, and climate. For instance, ecosystems are currently being modified by factors such as changes in the distribution of insect pollinators, which in turn are threatened by land‐use intensification, habitat destruction, pesticide use, climate change, invasive species, and the spread of diseases and parasites (Potts et al., 2010; Vanbergen et al., 2013). It is also widely accepted that climate change presents a serious threat to biodiversity because of changes in land use, fragmentation, and environmental degradation (Ackerly et al., 2010).

To gain insight from the perspective of environmental risk assessment, we conducted a national‐scale study of the relationships and potential for outcrossing between all the described Chilean vascular flora and species of agricultural interest. This was carried out using an improved outcrossing potential predictive tool called the total outcrossing potential (TOP) estimator, which allows for the establishment of outcrossing between cultivated species (including GM and non‐GM crops) and other cultivated crops and local flora. The TOP estimator considers pollinator insects, geographical, and climate factors involved in these donor–receptor interactions. The outcrossing potential estimator is presented as a part of an Internet resource (www.flujogenico.cl) that also includes several additional tools related to crop biotechnology in the country to improve the competitiveness of Chilean agriculture. This effort contributes to problem formulation as a first step in eventual ERAs for sustainable agricultural practices. This screening approach to problem formulation focuses on the likelihood of outcrossing to sexually compatible relatives of potential concern, such as those found between parallels 25°S and 40°S and the coast strip up to 19.5°S in the country. This study does not deal with the potential risks of introducing undesired traits (GM or non‐GM) to crops.

## 2 Methods

### 2.1 Informatics resources

For the platform design, open‐source software based on JavaScript was used. Several frameworks from MEAN Stack (MongoDB, Express.js, Angular.js, and Node.js.) were used for both the server and client sides. Server prototypes were programmed using Node.js, built on the JavaScript V8 Virtual Machine Engine from Google Chrome, and managed by the Node Package Manager (NPM). Express.js in Node.js was used as an extensible framework for HTTP server management based on Connect. Middleware was used to enable the provision of plug‐ins for the server. The databases are document oriented and have a non‐relational structure from MongoDB. The client prototypes use AngularJS for single‐page application design. Finally, workflow logs were registered using Git and Github.

### 2.2 Databases

#### 2.2.1 Vascular flora databases

The databases for native, introduced, cultivated, and GM plant species were described by Sánchez et al. (2016). Native and introduced species were updated by reviewing Zuloaga, Morrone, and Belgrano (2008) and considering the data status update by September 2016.

#### 2.2.2 Pollinator insect list

A bibliographic search was done using both printed and electronic information with baseline descriptions developed by Montalva and Ruz (2010). Insect groups were generated and organized hierarchically according to their relevance. A database was built with taxonomic information (order, genus, family, and species), vernacular names, diversity of plant species to be pollinated (polylectic or oligolectic for superfamily *Apoidea*), and whether they are endemic or introduced.

### 2.3 Algorithms

#### 2.3.1 Outcrossing potential index

The formula, factors, and conditions for calculation of the outcrossing potential index (OPI) have been described by Sánchez et al. (2016). Briefly, OP corresponds to a summarizing factor obtained for two species within a genus (i.e., pollen donor/pollen recipient), expressed as a percentage and built on the basis of the biological characteristics of each component of a donor–receptor couple. When an equivalence of the species is prompted (i.e., a conspecific hybridization is simulated), an additional score is immediately reflected by a minimum OP value of 50%. The OP values can be translated into a concept scale as indicated by Sánchez et al. (2016) and allow to express the outcrossing capability between these simulated species. The OP values do not consider geographical and climate conditions in the calculation and only refer to the biological conditions which could allow a hybridization between species in a genus.

#### 2.3.2 Regional pollinators index (RPI)

An index describing diversity and distribution of pollinator insects was built. Sectional latitude blocks were adopted as previously used for the superfamily *Apoidea* in Chile (Montalva & Ruz, 2010). These blocks corresponded to latitude areas from 18°S to 56°S (defining 18–19, 20–24, 25–29, 30–34, 35–39, 40–44, 45–49, 50–54, and 55–56 degrees south). Easter Island and Juan Fernández Archipelago were not considered. Blocks were used to describe insect taxa, and the block exhibiting the highest pollinator frequency (Pf) was used as the top reference (i.e., Pf_max_). An RPI_*i*_ associated with block *i* was built with the following formula:

(1) $${\text{RPI}}_{i}=\frac{{\text{Pf}}_{i}}{{\text{Pf}}_{\max}}$$

where Pf_*i*_ represents the pollinator frequency of block *i* and Pf_max_ represents the highest pollinator taxa frequency among blocks of the country.

The RPI was only considered in the case of modeling of entomophilous species. In the case of anemophilous species, the algorithm RPI is automatically cancelled and replaced by a numerical value = 1 in Formula (2) (see below), which represents that the influence of wind in the outcrossing will be considered at a maximum.

### 2.4 Species distribution probability

#### 2.4.1 Introduced and native species

An ecological niche model was developed for the occurrence probability of a pollen receptor species from these databases (both introduced and native). The focus of the model is establishing the relationship between species occurrence and the environmental variables characterizing the geographical locations in which those species occur. These relationships were used to project the occurrence of the same species to the complete region of the country by using an occurrence probability (Franklin, 2010).

As a data source for this model, two main sources were used: (1) geographic occurrence data for a subset of annotated species in the Chilean vascular flora databases (Sánchez et al., 2016), and (2) environmental variables derived using the WorldClim tool from a set of 19 climate layers (grids), which depend on the geographical characteristics of the country (Hijmans, Cameron, Parra, Jones, & Jarvis, 2005). From all of the species in the databases, a subset of 749 species (named “presence‐only” dataset) was modeled for SPD. These species included 282 introduced and 467 native and can be visualized at http://flujogenico.cl/index.php?option=com_phocadownload&view=category&id=14&Itemid=256. These species had a minimum of unique geographic locations per taxon of 30 records. The Arc‐Info workstation script (AML) described at http://www.worldclim.org/bioclim was also used. The bioclimate variables present annual climate trends and include maximal, minimal, and monthly average temperatures. These were weighted by geographical components such as slope and sun exposition. Distribution models were generated using Maxent software (Phillips, Anderson, & Schapire, 2006) using “default” parameters as indicated in Phillips and Dudík (2008), which gave a probability of occurrence per geographic unit at a maximum definition of 1 km^2^.

To compare species, occurrence probabilities were weighted and had a maximum species distribution probability (SDP) of one. Modeling and computing were carried out by CSW (Santiago, Chile; http://www.csw.cl).

#### 2.4.2 Cultivated species

The probability of occurrence for pollen receptor species from cultivated and GM databases was developed using the last agricultural census data (2007) obtained by the Oficina de Estudios y Políticas Agrarias Chile (ODEPA). The whole dataset available for the country up to the district level was considered. The probability of occurrence for each species was deduced as the ratio between the cultivated area (in hectares) for a specific species and the total cultivated area in that district. The probability of occurrence per geographic unit was defined up to 1 km^2^.

### 2.5 Total outcrossing potential

A TOP value linking OP, RPI, and SDP for a specific geographical point was built by the summation of these indexes using the following formula:

(2) TOP = OP×RPI×SDP

A geographical point corresponds to the user‐selected on‐screen point. This means coordinates (latitude, longitude) selected by the user on the map (i.e., on screen) in which a query is prompted in order to establish TOP calculations. Whereas OP depends on the species, geographical points will influence mainly on RPI and SDP.

### 2.6 Concept scale for TOP values

The TOP results were divided into quintiles (Q) for classification of the outcrossing potential as follows: Q1 (TOP = 0–0.20) = very low; Q2 = low; Q3 = medium; Q4 = high; and Q5 = very high.

## 3 Results

### 3.1 The datasets of the Web system

The Web tool consisted of three up‐to‐date plant databases previously described (Sánchez et al., 2016) and a new set dedicated to insects. In this latter, a total of 508 species associated with the pollination process were accessed, in which 98% (502) corresponded to endemic species and only six were introduced species, such as *Apis mellifera*,* Bombus terrestris*, and *Bombus ruderatus*. Out of all species, 438 corresponded to Hymenoptera (10 families and 80 genera), 40 to Diptera (7 families and 26 genera), 14 to Lepidoptera (5 families and 11 genera), and 14 to Coleoptera (10 families and 12 genera). The most frequently described native pollinators were the hymenoptera *Bombus dalhbomii* (Apidae), *Corynura chloris* (Halictidae), *Centris nigerrima*, and *Centris chilensis* (Apidae).

**Figure 1:**
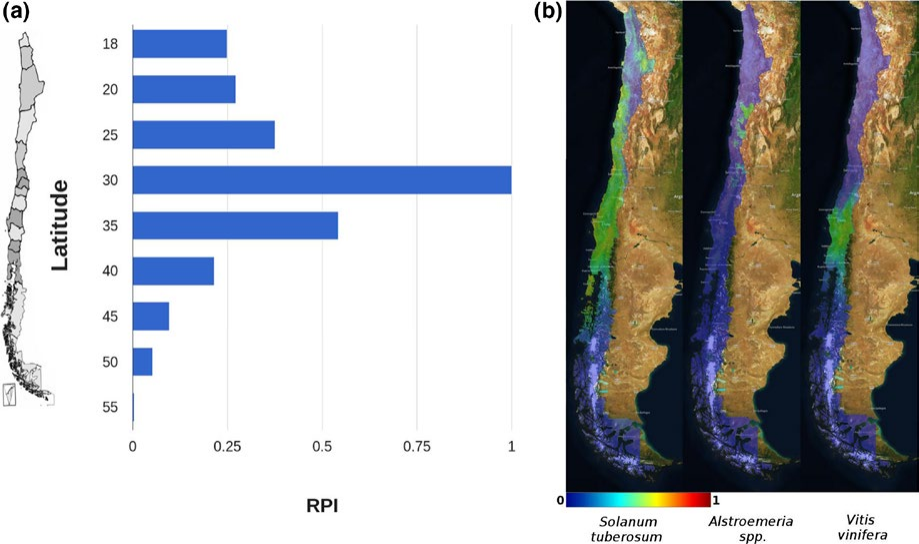
Frequency representation and system components. Pollinators (a) and vascular flora (b) data were organized and integrated into corresponding datasets. In the case of pollinators, distributions were calculated as a function of latitude (a). Vascular flora frequencies are represented as colors using a thermal color scale (from 0 to 1; b) using the “ecological niche model” for calculation of native and introduced species (with *S. tuberosum* and *Alstromeria* spp. as examples) or by a “probability of occurrence” for cultivated species defined by the ratio between the cultivated area for a specific species and the total cultivated area in that district (for *V. vinifera* in this instance)

The frequency of distribution describing pollinator agent occurrence showed a normal shape with most of the reported species (Figure 1a) between latitudes of 25° and 40° south (the north and central areas of the country), where the highest diversity of the Apoidea family of pollinators was found (Figure 1a). The complete dataset is available at http://epc.agroinformatica.cl/pollinators. In the case of vascular plant species datasets, patterns of distribution were deduced from their SDPs and varied depending on the type of species considered (cultivated, introduced, and native). Figure 1b shows example patterns of the distribution obtained for two representative native species, *S. tuberosum* and *Alstroemeria* spp., and one distribution pattern for the fruit crop *Vitis vinifera*. The complete list of vascular flora species considered can be found at http://epc.agroinformatica.cl/flora-vascular-chilena.

### 3.2 The outcrossing potential estimator tool

**Figure 2:**
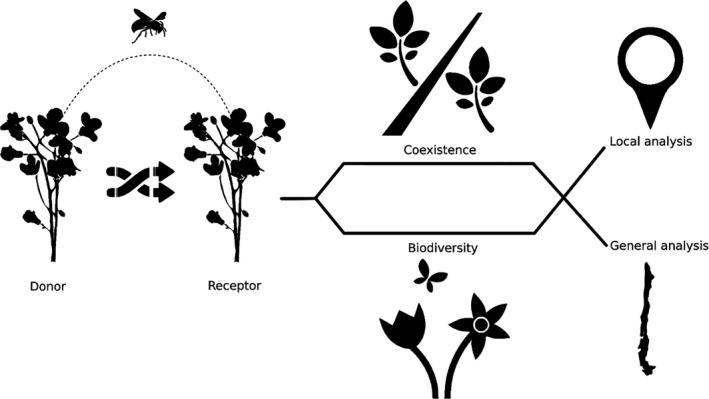
Flowchart of the total outcrossing potential (TOP) calculation. A donor species is selected for outcrossing potential (OP) evaluation, and the eventual donor–receptor interaction can be assumed as having coexistence (crop to crop) or biodiversity (crop to wild relatives) conditions. Once the condition is selected, either a national or a local scenario is selected. The effect on OP caused by the occurrence of pollinators and frequency of receptor species (considering climate and geographical conditions) is included in the local analysis

The TOP modeling tool defines two levels that consider the effect of introducing a cultivated or GM species (i.e., a pollen donor), as shown in Figure 2. The first refers to the biological scope of the donor–receptor interaction and considers two scenarios: (1) coexistence, in which species are located in consideration of a farming system such that outcrossings are understood between cultivated species, and (2) biodiversity, in which a cultivated (or GM) species is released into the environment, and the outcrossing is understood to proceed with native and introduced species. Once the scope is defined, a second level involving the geography of the simulation is incorporated using two scenarios: (1) nationwide, in which results are produced with a nationwide screening, and (2) local, in which results are produced by considering a limited region of the country. In the nationwide modeling, TOP values depend on the biological, botanical, and agronomical factors between donor and receptor species, regardless of geography. Values for TOPs using the local level also include the RPI, climate, and geographical factors.

### 3.3 Outcrossing potentials

#### 3.3.1 Vascular flora considerations

A grand total of 8,490 vascular species were computed. There were 256 Chilean cultivated species (i.e., those with evolutionary processes that have been influenced by humans to meet their needs). In this group, 157 species are of horticultural interest, 55 species are intended for ornamental purposes, and six species have forestry uses. The group of introduced flora was composed of 3,505 species and is also considered as a group of naturalized or exotic species. These plants are not native to a given area and instead have been accidentally or deliberately introduced by human activity. Finally, there were 4,993 native species, which are quantitatively the most important group.

#### 3.3.2 Coexistence situation: effect of cultivated on cultivated species

**Figure 3:**
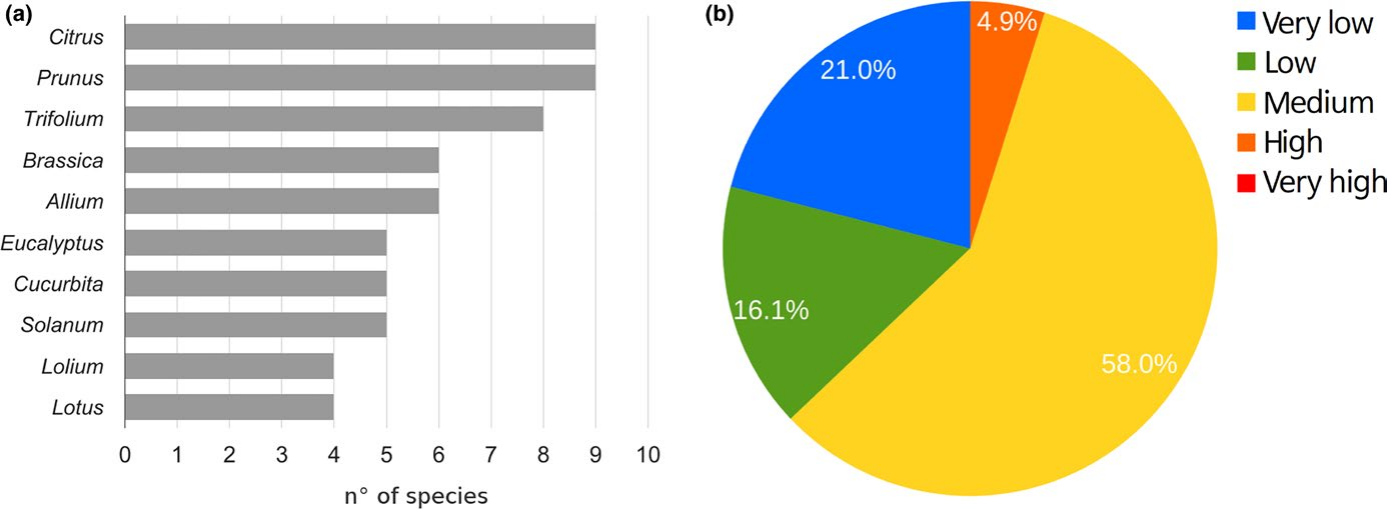
Effect of cultivated on cultivated species. The effect of cultivated species on the same group (i.e., coexistence) was calculated using total outcrossing potential (TOP). The genera with the most relative species (a) had between four and nine cultivated species. In terms of TOP values (b), the levels of outcrossing potentials were mostly in the medium, low, and very low ranges

The expected outcrossing between species belonging to the cultivated group showed that the 10 highest possibilities were found in species belonging to the genera *Citrus*,* Prunus*,* Trifolium*,* Brassica*,* Allium*,* Eucalyptus*,* Cucurbita*,* Solanum*,* Lollium,* and *Lotus* (Figure 3a). The number of species involved varied between 4 (for genera *Lotus* and *Lollium*) and 9 (*Citrus*). The highest outcrossing potential for species in this level fell in the range of Q4 (i.e., high risk) with 4.9% of the TOP values determined (Figure 3b).

#### 3.3.3 Biodiversity situation 1: effect of cultivated species on the local flora

Out of 256 cultivated species, just 7 (2.7%) corresponded to natives. At the genus level, the 7 cultivated‐native species showed 46 related native species. Most of the cultivated species in Chile were identified as introduced (249; 97.3% of the total cultivated); that is, the species itself is used as a crop. Two situations were observed in this cultivated‐introduced population: (1) a group in which the introduced species have no additional introduced relatives (52 species), and (2) the remaining 197 cultivated‐introduced species that have more than one relative. These cultivated‐introduced species with more than one relative reached 758 species of the introduced group, which belong to 101 different genera (21.6% of the total introduced vascular flora in the country).

**Figure 4:**
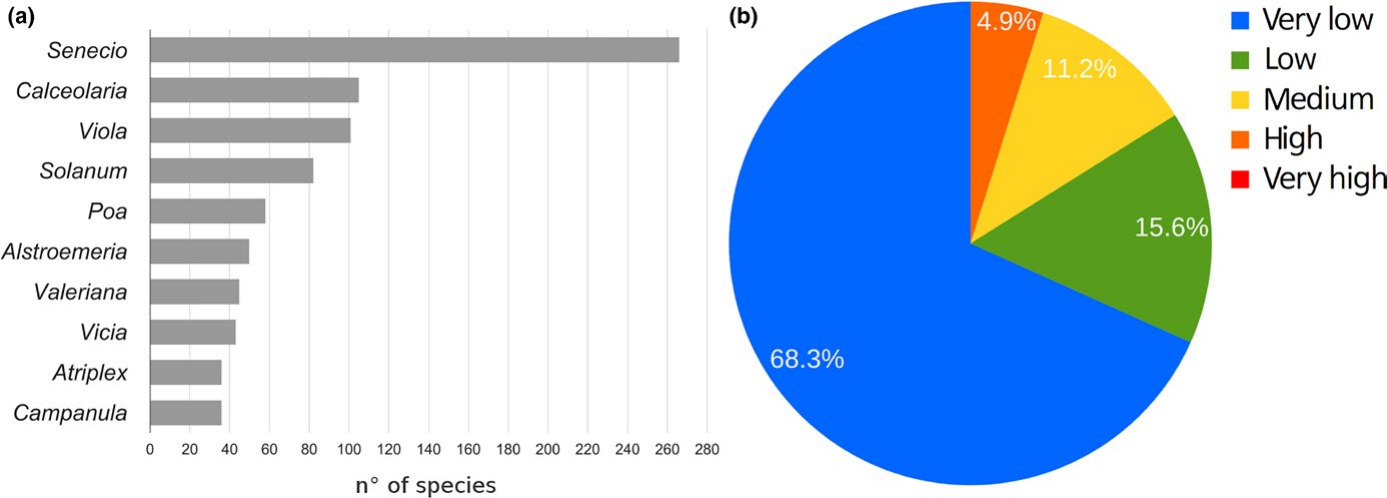
Effect of cultivated species on biodiversity. The effect of cultivated species on biodiversity (native plus introduced species) was calculated using total outcrossing potential (TOP). The genera with the most relative species (a) ranged from 36 to 266 native plus introduced species. In terms of TOP values (b), levels of outcrossing potentials were mostly very low

The expected outcrossing between cultivated species and local flora (native plus introduced species) showed that the 10 highest possibilities were found in the genera *Senecio*,* Calceolaria*,* Viola*,* Solanum*,* Poa*,* Alstroemeria*,* Valeriana*,* Vicia*,* Atriplex,* and *Campanula* (Figure 4a). After scoring all of the species in all of the genera, the number of species with outcrossing potential varied between 36 (for genera *Atriplex* and *Campanula*) and 266 (*Senecio*). The highest outcrossing potential for species at this level (genus) fell in the range of 0.60–0.80 (i.e., high risk), with 4.9% of the TOP values determined (Figure 4b).

#### 3.3.4 Biodiversity situation 2: effect of transgenic cultivated species on native species

**Figure 5:**
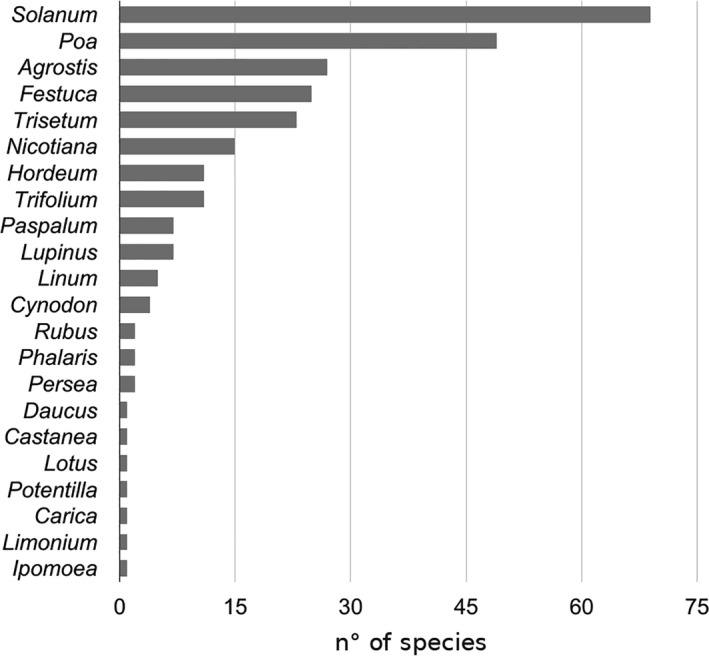
Transgenic species with native relatives. The effect of cultivated transgenic species on biodiversity (native plus introduced species) was calculated using TOP. The genera with the most relative species ranged from 1 (*Ipomea*,* Limonium*,* Carica*,* Potentilla*,* Lotus*,* Castanea*, and *Daucus*) to 66 native and introduced species (in the genus *Solanum*)

There were over 137 transgenic species included in the databases, representing 100 genera. There were 22 genera in the transgenic list that occur in Chile (30 species in total; Figure 5), which are related to 266 native species (5.3% of the total Chilean native vascular flora). Of these GM species, 78 taxa were introduced, including 25 that were naturalized (introduced species that have adapted and grow or multiply as if that were native), while 17 were classified as weedy, and 52 were conventional crops (cultivated). Among these species, the highest TOP values were obtained when the same species were coincident in both groups, such as those obtained for *C. chilensis*,* Prosopis tamarugo,* and *S. tuberosum*.

Outstanding TOP values were assigned to the species *Solanum pinnatum* (*syn. of S. albiforum*) and *S. gaudichaudii* when couples were formed with transgenic tomato, potato, and eggplant. Both native species are endemic with a restricted range of distribution and with serious conservation problems, as indicated by the National Cadastre developed by the National Ministry of the Environment (http://www.mma.gob.cl/clasificacionespecies/listado-especies-nativas-segun-estado-2014.htm). In the same analysis, *Solanum andinum* (*syn. of S. furcatum*) appeared to be sensitive to the same three transgenic species. *S. andinum* is endemic, is annual, and has a restricted range of distribution. Other *Solanum* species identified by this modeling included the endemic *S. phyllanthum (syn. of S. montanum)* and *S. remyanum*, which showed a non‐zero TOP value with the same transgenic species previously mentioned. Others groups of native species with TOP values over zero were *Agrostis* species, which are related to the transgenic *A. stolonifera* and *A. canina*. In the genus *Trifolium*, native species associated with the transgenic *T. repens* and *T. subterraneum* were *T. chilense, T. circumdatum, T. physantum,* and *T. vernum*, all of which are considered annuals and endemic.

Another important case identified within the results corresponded to the native species *F. chiloensis*, which showed TOP values corresponding to Q1. When its iteration was adjusted, there were three associated transgenic species: *F. vesca, F. virginiana,* and *Fragaria* sp. In the same way, a theoretical introduction of transgenic *Carica papaya* (papaya), *Linum usitatissimum*, and *Persea americana* in Chile should consider the existence of the native species *C. chilensis*,* Linum ramosissimum*, and *Persea meyeniana* in some regions of the country, all of which are endemic and are vulnerable species, according to the same National Cadastre.

At www.flujogenico.cl, “Supplementary table: Donor‐Receptor” in the “Downloads” section provides the complete set of iterations for donor–receptor couples, including all the cultivated (donor), native, and introduced (receptor) species, as well as their corresponding TOP values calculated by the Web tool. In addition, “Supplementary table: TOP Resume” provides all cultivated (donor) and receptor (native and introduced) genera, the number of involved species, and the average TOP values by genus.

## 4 Discussion

Agriculture, including the seed industry, involves genetically uniform and dominating species in defined landscapes. Consequently, these highly dense productive spots are expected to have very different levels of impact on themselves and their surrounding environments (Butler, Vickery, & Norris, 2007). Effective management plans and actions in agriculture can only be achieved after acquisition of demography and the collection of spatial data, the latter of which includes both weather and other tightly associated elements such as pollinator insects (Picó, Rodrigo, & Retana, 2008). We have introduced a modeling tool that predicts outcrossing potentials between species with additional fundamental considerations of weather, pollinator insects, and productive status of the species in the different ecosystems.

Diverse computer systems addressing different agriculture aspects have been developed over the past decade (Holzworth et al., 2015). These have focused on both productive and strategic issues, such as climate change and adaptation (White, Hoogenboomb, Kimballa, & Walla, 2011), resource use and efficiency (Salazar et al., 2012), plant breeding (Hoogenboom, White, & Messina, 2004), and pests and diseases (Garrett, Dendy, Frank, Rouse, & Travers, 2006), among others. Also, species distribution and ecological niche models have been applied and improved to address similar concerns in biodiversity and ecology (Mesgaran, Cousens, & Webber, 2014; Slavich, Warton, Ashcroft, Gollan, & Ramp, 2014). The current trend of using and generating cross‐referenced networks is made possible today by powerful programming tools that allow for faster computing and displaying the results (Holzworth et al., 2015). In this way, the Web platform is based on the V8 JavaScript engine, which is widely used open‐source software. The system also includes available NPM code packages (such as node.js and angular.js) to facilitate crosstalk, side applications, and up‐to‐date information. The system was also built by incorporating freely available information from dedicated servers and tools, such as WorldClim.

The impact of a crop on its environment is clearly marked by historical aspects of the environment (Rufener, Mazyad, & Ammann, 1999; Tufto, 1999). In the case of Chile, those aspects refer to a geographically insulated region with wide climate heterogeneity, which has made it a very important area of plant biodiversity (Zuloaga et al., 2008). There are almost 5,100 species, of which 51.5% are considered as endemic (Marticorena, 1990; Zuloaga et al., 2008). The main zone recognized as a center for diversification and speciation is located between parallels 25°–40° and in the coastal string up to 19.5°. Interestingly, the database of pollinator insects and their distribution (Figure 1a) correlates these observations and shows the eventual distribution of relevant pollinators according to the distributions of plant populations. In areas of sympatry, both plant‐specific traits and ecological attributes of the environment can affect pollinator movement. Thus, pollen and nectar are important attractants that can influence pollinator visitation rates and plant mating success (Hersch & Roy, 2007).

Estimation of plant hybridization can vary dramatically among regions and sources, and it has been suggested that hybridization behavior of a species group (family or genus) may be determined more by its intrinsic properties than by environmental conditions (Whitney et al., 2010). No major differences can be expected between GM and cultivated species in several factors, including fertility and fitness (Sweet & Bartsch, 2012), but not cases in which there is an *ex professo* modification of sexual traits, such as andro‐sterility (Schnable & Wise, 1998). Crops and wild relatives of the same species (and even genus) can cross‐pollinate, depending on biological and environmental characteristics such as phylogenetic closeness, sexual compatibility, geographical localization (distances), population composition and densities, pollinator agents, and climate (temperature, humidity, wind direction).

In addition, pre‐pollination (pollen emission, dispersion, and reception) and post‐pollination (fertilization) events must be considered in the analyses. Chile is a center of origin or diversity for important cultivated species like *S. tuberosum* sp. *tuberosum*,* Phaseolus vulgaris,* and *Zea mays*. Furthermore, some wild relatives such as *F. chiloensis, Alstroemeria* spp*.,* and *Bromus* spp. can be found as crops that share the same areas. There are also about 2,500 species that have been classified as introduced, and some of them have become naturalized or even weedy species in some cases (Espinoza, 1996; Matthei, 1995; Zuloaga et al., 2008). Together with these botanical characteristics, a remarkable aspect of this new Web tool results from the use in local analysis mode of relevant conditions found in the different ecological niches evaluated, which include the geographical conditions and climate of a habitat.

Several regional‐scale studies have established eventual hybridization rates between crop species and native flora (Whitney et al., 2010). Relevant data have been produced for the UK (Raybould & Gray, 1993), Ireland (Flannery et al., 2005), the Netherlands and Switzerland (de Vries, van der Meijden, & Brandenburg, 1992), and New Zealand (Armstrong et al., 2005). In the case of Chile, outcrossing potentials using agronomical and geographical distributions of species have indicated that no major species would be affected by the inclusion of the 11 most common GM crops used worldwide (Sánchez et al., 2016). Results have demonstrated that Chilean environments are less impacted by commercial species planted than previously thought (Sánchez et al., 2016).

Chile is an important area for high‐quality seed production activity, ranking sixth for seed exports worldwide (ISF, 2013). The activity also incorporates GM seed production, which is subjected to a local regulatory framework that exclusively involves multiplication and re‐export. The most relevant GM crops in the country are maize, soybean, and canola. In the present work, the extension of the analysis toward the total number of cultivated species in the country showed that almost 20% of the native and introduced species have relatives in those groups (Figures 3 and 4). In addition, this rate was reduced into 5% of the native species when transgenic species were modeled as impacting the native Chilean species (Figure 5).

In this way, although the current results confirm previous analyses, they also indicate that certain considerations should be made with special emphasis on genera such as *Solanum*,* Poa*,* Agrostis*,* Festuca*,* Tricetum*,* Lupinus*, and a few others, which could also be of agricultural interest in specific areas in the country. Whereas ecologists define species diversity based on species richness (the number of species) and species evenness (their relative abundance), mapping areas with high or specific plant biodiversity represent a priority for decision makers, and the Web platform and TOP predictor could be important supporting tools for both areas.

The use of more powerful computers, programming languages, and the Internet access has resulted in considerable contributions to this new tool for Chilean vascular flora and their relationships with modern agriculture. In addition, outcrossing potential results between species can now be modeled. The occurrence of these interactions can also be judged based on spatial and climate information, which could lead to case‐by‐case analysis of the actual possibility of events taking place in the country.

## Author Contributions

H.P. conceived the general idea of the system, databases structure, and calculation tools. P.C., D.Z., M.M., and E.S. developed vascular flora databases. P.C. and H.N. developed pollinators database. P.C. and M.R. conducted in silico outcrossing potential analyses. P.C., C.A., G.C., D.Z. conducted programming projects. P.C., H.P., and M.S. analyzed data. H.P. wrote the manuscript. All authors read and approved the manuscript.

## Conflict of Interest

Authors declare no competing interests as defined by the Journal, or other interests that might be perceived to influence the results and/or discussion reported in this article.
